# Application of Surface Complexation Modeling to Investigate the Mechanism of Cu^2+^ Adsorption on TiO_2_, Al_2_O_3_, and SiO_2_ Under High Surface Coverage

**DOI:** 10.3390/molecules29235595

**Published:** 2024-11-26

**Authors:** Wojciech Piasecki, Karolina Lament

**Affiliations:** Department of Physical Education and Health, Józef Piłsudski University of Physical Education in Warsaw, Akademicka 2, 21-500 Biała Podlaska, Poland; karolina.lament@awf.edu.pl

**Keywords:** copper adsorption, metal oxide, surface complexation modeling, hydrogen surface charge, electrokinetic potential, Cu-ISE

## Abstract

We have shown that the adsorption of Cu^2+^ ions on various metal oxides, depending on the pH of the solution, can be described assuming the formation of only two surface complexes with surface hydroxyl groups SOH: SOCu(OH) and SOCu^+^. Using an ion-selective electrode for Cu^2+^, we determined the adsorption edges, i.e., the dependence of the amount of adsorbed metal expressed as a percentage depending on the solution pH for three oxides: TiO_2_, Al_2_O_3_, and SiO_2_. The measurements were carried out with high surface coverage where the ratio of the adsorption sites/copper ions in the system were from 2 to 3, depending on the oxide. Simultaneously, with the adsorption edge, the hydrogen surface charge density and the electrokinetic potential of the oxide particles were measured as a function of pH. These three types of experimental data were fitted all together using the surface complexation model (2-pK TLM). In modeling, it was not necessary to consider the precipitation of Cu(OH)_2_ on the oxide surface to obtain good agreement with the data. Additionally, it was shown that the presence of charged surface species SOCu^+^ (about 10% of total adsorbed copper) was crucial to fit the data for zeta potential.

## 1. Introduction

Water pollution with heavy metals is one of the most serious problems in environmental protection [1]. The main sources of these pollutants are industry and mining. Copper is the most used metal in the economy, apart from iron and aluminum. Unlike iron and aluminum, copper is not commonly found in the minerals that make up the Earth’s crust. The aqueous concentration of major components like iron is controlled by solubility equilibria, whereas the concentration of trace elements like copper is controlled by sorption equilibria [2].

The appearance of copper ions in high concentrations in surface waters indicates their contamination is of an anthropogenic origin. Taking into account toxicological properties, copper is considered a microelement in low concentrations but a highly toxic element in high concentrations [3]. In aqueous systems, copper toxicity depends on the solution’s concentration of free metal ions. For example, the concentration of copper in drinking water should not exceed 1.3 mg·dm^−3^ [4].

Many methods have been developed to remove various contaminants from water [1,5,6,7,8,9]. The most effective and least expensive methods are those based on the adsorption process. The most used adsorbents are activated carbons obtained from various organic wastes and inorganic nanomaterials [10,11]. The latter group includes metal oxides. The most widely used in industry are titanium, aluminum, silicon, and iron oxides. These materials can be cheaply produced as nanometric particles with a very large specific surface area [4,12]. Such particles in water are characterized by a remarkably high affinity for metal ions.

In the literature on the adsorption of divalent metal ions, measured adsorption isotherms are analyzed using classical Langmuir or Freundlich equations, which are not based on the molecular mechanism of this process [4,10]. It should be emphasized here that it is crucial to consider the variable surface charge of oxides in the theoretical description of ion adsorption from a solution.

In water, the surface of the oxide is covered with hydroxyl groups SOH (S here denotes the surface). These groups can attach hydrogen ions or dissociate them, thus acquiring an electric charge, which depends mainly on the pH of the solution and the number of adsorbed ions. Surface hydroxyl groups can bind metal ions on the oxide surface as ligands. These processes are often called surface complexation [13,14]. Theoretical models describing the ion adsorption process on the oxide surface as a complex formation reaction are briefly referred to as SCMs (surface complexation models) [15]. In these models, it is essential to determine the location of the adsorbed ions, i.e., their distance from the charged oxide surface. Therefore, additional layers in which different ions are located are introduced into the structure of the electric double layer formed in solution on the oxide surface. One of the most popular models of this type is the 2-pK TLM (Triple Layer Model) [14]. In this model, ions can adsorb directly on the oxide surface, forming inner-sphere complexes, or at a certain distance from the surface, forming outer-sphere complexes, or being in the diffusion layer, where only weak electrostatic interactions of ions with the charged surface occur. Additionally, surface charging may occur due to proton association or dissociation reactions (2-pK protonation mechanism).

To correctly model using SCMs, we must first have information about the structure of the complexes formed on the surface and the mechanism of the adsorption process. Such information can be provided by spectroscopic measurements such as EXAFS and ion adsorption kinetics studies [16]. Bochatay et al. investigated Cu(II) adsorption on the water-goethite (α-FeOOH) interface using EXAFS [17]. They found that copper ions formed inner-sphere surface complexes on goethite. They could not determine how exactly Cu^2+^ ions are coordinated to the surface. However, their measurements have indicated that at pH 8, Cu^2+^ could form hydroxo-bridged polymeric species on the surface. Using EXAFS spectroscopy and molecular modeling, Peacock and Sherman studied Cu(II) sorption on three ferric oxides: hematite (α-Fe_2_O_3_), goethite, and lepidocrocite (γ-FeOOH) [18]. They identified bidentate and tridentate surface complexes of copper ions on the surface. They also excluded the formation of surface precipitates of Cu^2+^.

Another direction of research into the ion adsorption mechanism is kinetic studies based on relaxation techniques, which consist of a sudden disturbance of the equilibrium state in the system by, for example, a pressure jump, and then tracking the changes in ion concentration as they reach a new equilibrium position [19]. In this way, Chang et al. studied the adsorption of copper ions on alumina (γ-Al_2_O_3_) [20]. They found that it is a two-step process. First, a proton is released from the SOH group. Then Cu^2+^ ions react with the negatively charged ${SO}^{-}$ group, forming an inner-sphere surface complex $SOCu^{+}$. The same technique was used by Grossl et al. to study the kinetics of Cu(II) adsorption on goethite [21]. They found that the adsorption rate of divalent ions on the oxide directly depends on the rate of a water molecule removal from the first hydration layer of the adsorbing ion, which indicates the formation of inner-sphere surface complexes.

In the literature, one can most often find measurements of the adsorption isotherm of copper ions on oxides, which are then fitted using the Langmuir, Freundlich, or other classical adsorption isotherm equation [4,12]. Less frequently, one can find measured adsorption edges, i.e., the dependence of the percentage of adsorbed ions on the pH of the solution [22]. To fit the latter data, one has to use the oxide surface complexation model [23]. On the other hand, one can very rarely find publications where, apart from ion adsorption, other quantities that change during the adsorption of divalent metal ions, such as the surface charge and electrokinetic potential of the oxide particles, were also measured [24,25].

In this publication, we would like to show for the first time the adsorption edges for copper ions determined using an ion-selective electrode and the simultaneously measured changes in the hydrogen surface charge density as well as the values of the electrokinetic potential. These measurements were performed for three different oxides: titanium dioxide ($TiO_{2}$), aluminum oxide ($Al_{2}O_{3}$), and silicon dioxide ($SiO_{2}$). We have already studied these three oxides before [24,25]. Our work aims to propose a simple model that would describe the different data recorded during the adsorption of copper ions on various oxides.

## 2. Results

Many researchers measuring the adsorption isotherms of divalent ions seem to forget that binding these ions to the oxide surface is accompanied by the release of significant amounts of hydrogen ions into the solution, which decreases the solution pH [26]. Moreover, this phenomenon also applies to other adsorbents, such as activated carbons, which have active groups on their surface that are proton donors [11].

Ensuring pH constancy during adsorption measurements is not trivial. Common buffers are often excluded because they are not inert electrolytes. Either manual or automatic pH correction during adsorption is required. Therefore, the commonly determined adsorption isotherm for adsorption on oxides may not be the optimal choice.

The key parameter that determines the adsorption properties of the oxide surface is the pH of the solution in contact with this surface. Therefore, a highly effective method of studying the adsorption of metal ions is to determine the so-called adsorption edge, which is measured by introducing a portion of metal ions into the acidified suspension of the oxide and gradually increasing the pH of the solution. After each base addition, the suspension is stirred, and the pH and metal concentration stabilize. Then, we measure the concentration of the metal ion in the solution.

**Figure 1:**
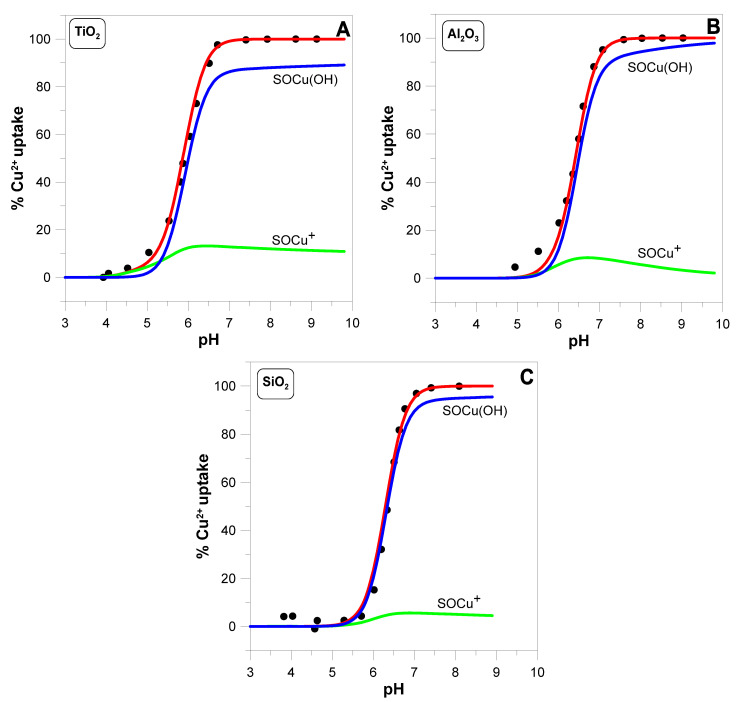
The adsorption edges of $Cu^{2+}$ ions (initial concentration 1 mM) on $TiO_{2}$ (**A**), $Al_{2}O_{3}$ (**B**), and $SiO_{2}$ (**C**) as a function of pH measured in 0.1 M $KNO_{3}$ solution containing 2 g·dm^−3^ oxide. Theoretical predictions (red lines) were based on surface reactions (1a–d) and (3a,b). The contributions from the surface species $SOCu^{+}$ (green line) and SOCu(OH) (blue line) are also shown. The values of the parameters used in the calculations are summarized in Table 1.

The results are presented as a graph showing the percentage of adsorbed ions (or the percentage of its uptake) as a function of pH (see Figure 1). By measuring the adsorption edge, we can also precisely control the balance of hydrogen ions on the oxide surface and determine the so-called hydrogen (proton) surface charge density $\sigma_{H}$ (see the Materials and Methods in Section 4). Additionally, during the measurement, we can collect small samples of the suspension and determine the electrokinetic potential of the oxide particles. Thus, during one experiment, we independently determined three quantities: percentage of adsorption, hydrogen surface charge density $\sigma_{H},\;$and ζ-potential. Such a data set allows for reliable modeling of the adsorption process of divalent ions on oxides.

An important issue is measuring the concentration of metal ions in the oxide suspension in successive titration steps. This can be achieved using classical spectroscopic techniques, but it is multi-step and laborious. The solution to this problem may be using ion-selective electrodes (ISEs). The primary condition here is the possibility of using such an electrode in a wide range of pH values. An electrode with these parameters is Cu-ISE from Metrohm, which we used to determine the concentration of copper ions [27].

We conducted the copper ion adsorption study for three different oxides: $SiO_{2}$, $TiO_{2}$, $Al_{2}O_{3}$. These oxides differ significantly in their acidity. Their point of zero charge (PZC), i.e., pH at which the charge on the oxide surface is zero, is, respectively, 3–4, 6.6, and 8.8 [24,25] (where PZC for silica cannot be precisely determined).

In our experiments, we added 0.2 g of oxide and 0.1 mmol of copper ions to a titration vessel containing 100 cm^3^ 0.1 M $KNO_{3}$ solution (inert electrolyte). Knowing the specific surface area of the oxides and the number of adsorption sites (SOH groups) per unit surface area, we can calculate the total number of moles of surface hydroxyl groups for each oxide. These values are 0.21 mmol for $TiO_{2}$, 0.27 mmol for $Al_{2}O_{3}$, and 0.31 mol for $SiO_{2}$, respectively. The given values should be considered approximate because the specific surface area of the oxide is determined with a significant error (about 20%). It is easy to see that the ratio of the number of moles of available SOH groups to the number of moles of copper ions is from 2 to 3. Therefore, $Cu^{2+}$ adsorption occurs in the range of high surface coverage.

Such a ratio of the oxide to copper ions was not accidental. Adsorption edges or isotherms can be determined for very small concentrations of metal ions if we have a suitably sensitive method of detecting these ions in solution. In the case of surface charge and ζ-potential, the influence of exceedingly small amounts of adsorbed divalent metal ions on these quantities may be practically unnoticeable. This means that these two quantities cannot be used to determine the adsorption mechanism in such a case.

Figure 1 shows the adsorption edges of copper ions at a concentration of 1 mM on three metal oxides at a concentration of 2 g·dm^−3^ determined using an ion-selective electrode for $Cu^{2+}$. In the case of $TiO_{2}$, copper adsorption begins at pH 4 and ends at pH 7. For $Al_{2}O_{3}$, adsorption begins at pH 5 and ends at pH 8. For silica, the edge is the steepest because it begins at pH 5.5 and ends at pH 7.

The obtained experimental data were modeled using the Geosurf code developed by Sahai and Sverjensky [28]. This program has been successfully used to model the adsorption of various divalent ions on oxides [29,30]. The Geosurf code was based on the 2-pK TLM model of the electrical double layer formed at the metal oxide/electrolyte solution interface [15]. This model assumes that the surface SOH groups are amphoteric and can either attach or dissociate protons. Additionally, it is believed that the inert electrolyte ions can form outer-sphere complexes on the surface in the form of ion pairs. The set of the above surface reactions can be written as follows:

(1a) $$SOH+H^{+}\to SOH_{2}^{+}$$

(1b) $$SOH\to SO^{-}+H^{+}$$

(1c) $$SOH+K^{+}\to SO^{-}{\_K}^{+}+H^{+}$$

(1d) $$SOH+H^{+}+NO_{3}^{-}\to SOH_{2}^{+}\_NO_{3}^{-}$$

Each of these reactions is described by an equilibrium constant, which, for example, for reaction (1a), has the form:

(2) $$K_{1}=\frac{\left[SOH_{2}^{+}\right]}{\left[SOH\right]a_{H}}\exp\left(\frac{e\psi_{0}}{kT}\right)$$

where square brackets denote the concentration of surface species, $a_{H}$ is a hydrogen ion activity in solution, $\psi_{0}$ is electric potential on the surface of oxide, and the symbols *e*, *k*, and *T* have their standard meaning. The exponential term is the Boltzmann factor, which corrects hydrogen ion activity at the surface relative to the bulk phase.

To link the electric potential with the surface charge in the 2-pK TLM, two electric capacitances, $c_{1}$ and $c_{2}$ are introduced. The higher capacitance value means that the ions in the layer described by it are closer to the oxide surface.

To describe the adsorption of copper ions on the oxides, we must assume the structure of the surface complexes formed. In this work, we have assumed that copper forms the following surface species: $SOCu^{+}$ and SOCu(OH). These are inner-sphere complexes, where copper ions are located directly on the oxide surface (where the potential is $\psi_{0}$). Robertson and Leckie successfully used these two complexes to describe the adsorption of copper on goethite [26]. The reactions below show the formation of these surface species:

(3a) $$SOH+Cu^{2+}\to SOCu^{+}+H^{+}$$

(3b) $$SOH+Cu^{2+}+H_{2}O\to SOCu\left(OH\right)+2{\;H}^{+}$$

Figure 1 shows how the complexes proposed in reactions (3a,b) reproduce the measured adsorption edges. The red line indicates the calculated total adsorption, while the green and blue lines represent the contributions from the surface species $SOCu^{+}$ and SOCu(OH), respectively.

**Figure 2:**
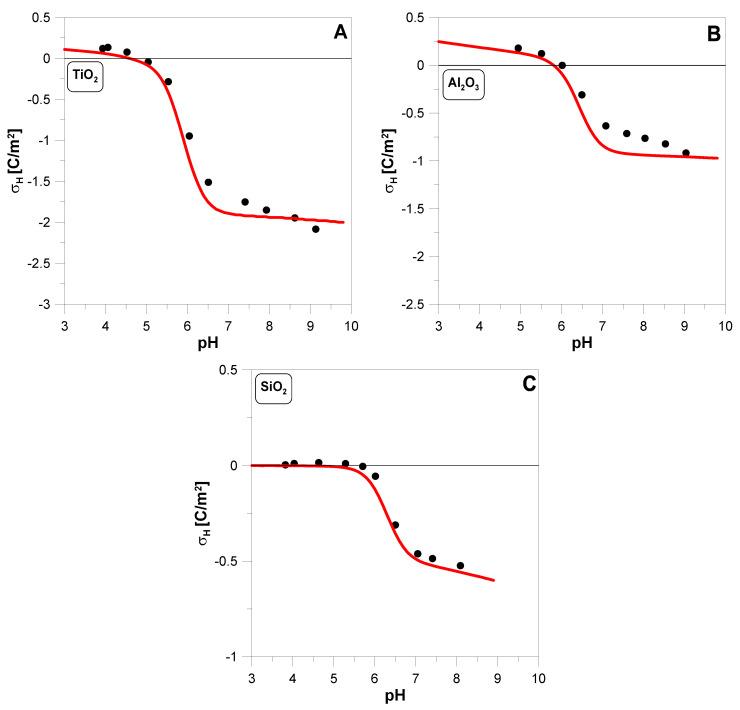
The hydrogen surface charge density *σ_H_* on $TiO_{2}$ (**A**), $Al_{2}O_{3}$ (**B**), and $SiO_{2}$ (**C**) as a function of pH measured in 0.1 M $KNO_{3}$ solution containing 2 g·dm^−3^ oxide. Theoretical predictions (red lines) were based on surface reactions (1a–d) and (3a,b). The values of the parameters used in the calculations are summarized in Table 1.

Figure 2 shows the hydrogen surface charge density $\sigma_{H}$ determined for three oxides during the adsorption of copper ions. It is worth noting that a significant decrease in the $\sigma_{H}$ value is observed in the pH ranges where the adsorption edges are located. Considering Equation (3a,b), this is an understandable phenomenon.

**Figure 3:**
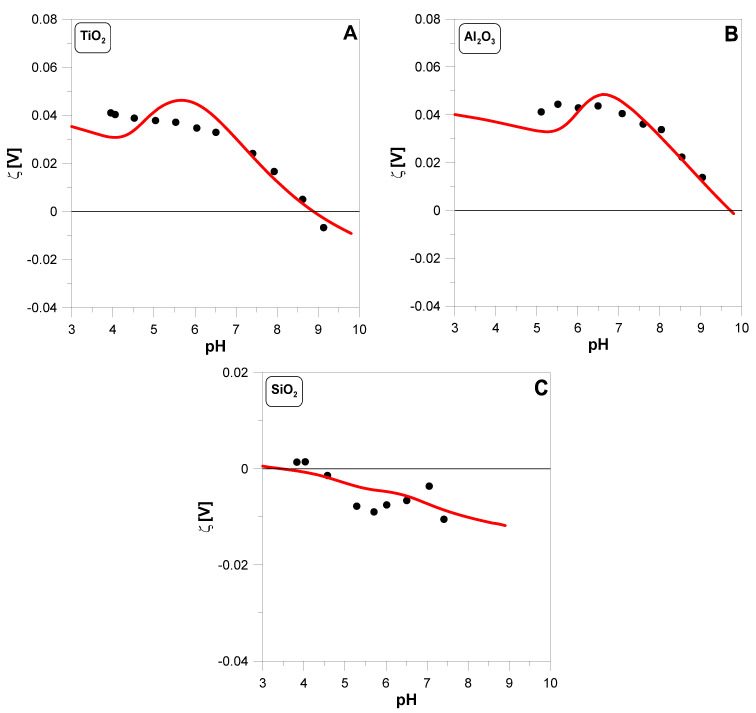
The ζ-potential of $TiO_{2}$ (**A**), $Al_{2}O_{3}$ (**B**), and $SiO_{2}$ (**C**) particles as a function of pH measured in 0.1 M $KNO_{3}$ solution containing 2 g·dm^−3^ oxide. Theoretical predictions (red lines) were based on surface reactions (1a–d) and (3a,b). The values of the parameters used in the calculations are summarized in Table 1.

In Figure 3, we can see the electrokinetic potential measured for three oxides. In the case of $TiO_{2}$ and $Al_{2}O_{3}$, it is shifted towards positive values. The quality of the data fit is satisfactory. Some discrepancies occur only for low pH values.

The equilibrium constants of the surface reactions and other parameters are collected in Table 1.

## 3. Discussion

It is commonly believed that divalent ions adsorbed on the oxide surface may hydrolyze [2]. This is because the concentration of these ions at the surface is higher than in the solution, and the concentration of hydrogen ions is lower there. Reaction (3b) shows the formation of such a hydrolyzed complex for copper. Ezati et al. calculated the speciation of copper in solution in the presence of metal oxides. They found that free copper ions are the predominant form in solution at pH values from 4.0 to 6.5 [4].

It should be recalled here that the formation of the $SOCu^{+}$ complex described by reaction (3a) was confirmed by the analysis of kinetic data for the adsorption of $Cu^{2+}$ on alumina and goethite [20,21]. Additionally, reaction (3b) can be treated as a complex reaction, where reaction (3a) occurs first, and then the formed surface species $SOCu^{+}$ undergo hydrolysis and finally SOCu(OH) is formed.

The stoichiometry of the surface complex formation reaction is essential in the theoretical analysis of ion adsorption. Let us now analyze the reactions (3a,b). According to the first one, the adsorption of 1 mole of $Cu^{2+}$ ions is accompanied by the release of 1 mole of $H^{+}$ ions, and the surface becomes positively charged. The second reaction shows that the binding of 1 $Cu^{2+}$ ion on the surface is accompanied by the release of 2 $H^{+}$ ions, and the oxide surface does not gain any additional charge. The more hydrogen ions participate in a reaction, the more sensitive it is to pH changes. This is manifested by a steeper course of the adsorption edge. In turn, increasing the number of protons released during the adsorption of copper ions causes the hydrogen surface charge density to decrease. The above observations are essential when analyzing the hydrogen surface charge and ζ-potential data.

Figure 1 shows theoretical predictions (red lines) of copper adsorption edges for three oxides. The electrically neutral, hydrolysed SOCu(OH) complex clearly dominates. The share of positively charged $SOCu^{+}$ species is about 10%. This small proportion of positively charged copper complex may significantly impact the electrokinetic potential.

In Figure 2, we see that the fit of the hydrogen charge by our model (red line) for titanium dioxide and alumina may not be perfect, but it reproduces the course of the experimental points well. The clear difference between the experiment and theory is observed in the pH range of 7–8 for alumina. This is a strange phenomenon because for pH > 7, almost all the copper is already adsorbed on the oxide, and it is not easy to find a process responsible for this behavior. In the case of silica, the decrease in $\sigma_{H}$ is much smaller than in the case of the other two oxides. This is because the silica surface in the tested pH range is already strongly deprotonated.

Figure 3 shows a good fit between the theoretically calculated ζ-potential and the experimental data. The role of the $SOCu^{+}$ species must be emphasized here.

A small amount of the $SOCu^{+}$ complex increases the positive charge on the surface of the oxide particle; therefore, in the case of $TiO_{2}$ and $Al_{2}O_{3}$, the zeta potential is positive in almost the entire measured pH range. In the case of $SiO_{2}$, there are many negatively ionized $SiO^{-}$ groups on the surface and the appearance of the positively charged $SOCu^{+}$ complex does not significantly affect the total charge.

In one of our previous papers, we studied the adsorption of calcium ions on oxides [25]. $Ca^{2+}$ ions adsorb much weaker than $Cu^{2+}$ ions. Their adsorption on $Al_{2}O_{3}$ and $SiO_{2}$ begins above pH 7 and even at pH 10 does not exceed 60%. The mechanism of $Ca^{2+}$ binding to the oxide surface is also different than that of $Cu^{2+}$. Calcium ions form outer-sphere complexes, while copper ions are closer to the surface and form inner-sphere complexes.

At least several publications have been devoted to modeling the adsorption of ions on oxides with high surface coverage [31,32,33]. Katz and Hayes were the first to propose several methods for describing the adsorption of cobalt ions on alumina at high surface loading [31]. According to them: “The solid solution model accounts for high coverage data by invoking a coprecipitation surface reaction, while the polymer model accomplishes the same task with multinuclear surface complexation reactions. In the continuum model, two polymer reactions and one precipitation reaction are proposed” [31]. Ultimately, they chose the continuum model as their preferred model because its predictions best matched the spectroscopic data.

Based on the ideas proposed by Katz and Hayes, Subramaniam et al. conducted a theoretical analysis of the collected data [33]. They analyzed the adsorption of copper ions on iron oxide and silica and monitored the changes in the electrokinetic potential during adsorption. They found that the classical SCM is sufficient to describe the adsorption of $Cu^{2+}$ on iron oxide if copper forms outer-sphere complexes with the structure $SO^{-}Cu{\left(OH\right)}^{+}$. In the case of silica, it was necessary to use the surface polymer model or the continuum model to correctly describe the data. Subramaniam et al. did not measure the change in surface charge caused by copper adsorption. They only determined the charge of oxides in the inert electrolyte to calibrate their model. They also did not measure the concentration of copper ions using an ion-selective electrode.

Karthikeyan and Elliott applied the generalized two-layer model to describe copper sorption by hydrous oxides of iron and aluminum [32]. According to them, at high pH and a high sorbate/sorbent ratio, in addition to adsorption, the coprecipitation of copper should also be considered.

Christl and Kretzschmar investigated the influence of the assumed value of the surface site density on the modeling of experimental data of copper and lead ions adsorption on hematite using the 2-pK basic Stern model [34]. The solid concentration was 2 g·dm^−3^, and the maximum metal concentration was 0.1 mM. They found that the surface site density is insignificant for modeling single-metal sorption data but becomes an essential parameter for describing multicomponent systems. Competitive sorption of $Cu^{2+}$ on $TiO_{2}$ was also investigated by Konstantinou and Pashalidis by applying Cu-ISE [35]. Ludwig and Schindler also used Cu-ISE to monitor the adsorption of copper ions on titanium dioxide [22]. In turn, Juang and Wu monitored the changes in ζ-potential caused by the adsorption of copper and sulfate ions on goethite [36].

The most in-depth studies of the mechanism of copper ion adsorption on three iron oxides were performed by Peacock and Sherman [18]. They found that neither copper adsorption isotherms nor EXAFS spectra supported the formation of surface precipitates on iron oxides although we are in supersaturated region for CuO and $Cu{\left(OH\right)}_{2}$. Based on spectroscopic data and ab initio molecular geometry calculations, they proposed the formation of the following bidentate and tridentate surface complexes of copper ions [18]:

(4a) $$2SOH+Cu^{2+}+2H_{2}O\to {\left(SOH\right)}_{2}Cu{\left(OH\right)}_{2}+2H^{+}$$

(4b) $$3SOH+2Cu^{2+}+3H_{2}O\to {\left(SOH\right)}_{2}SOCu_{2}{\left(OH\right)}_{3}+4H^{+}$$

**Figure 4:**
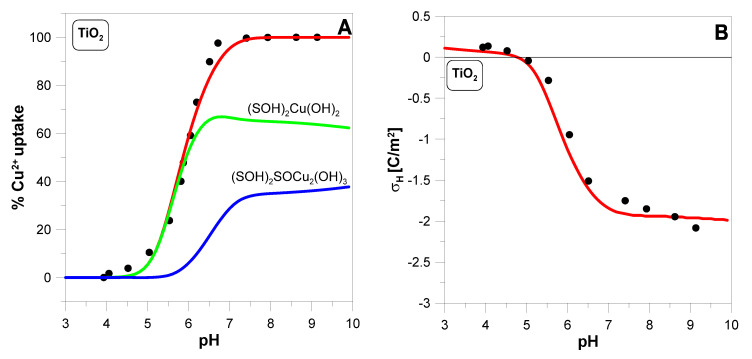

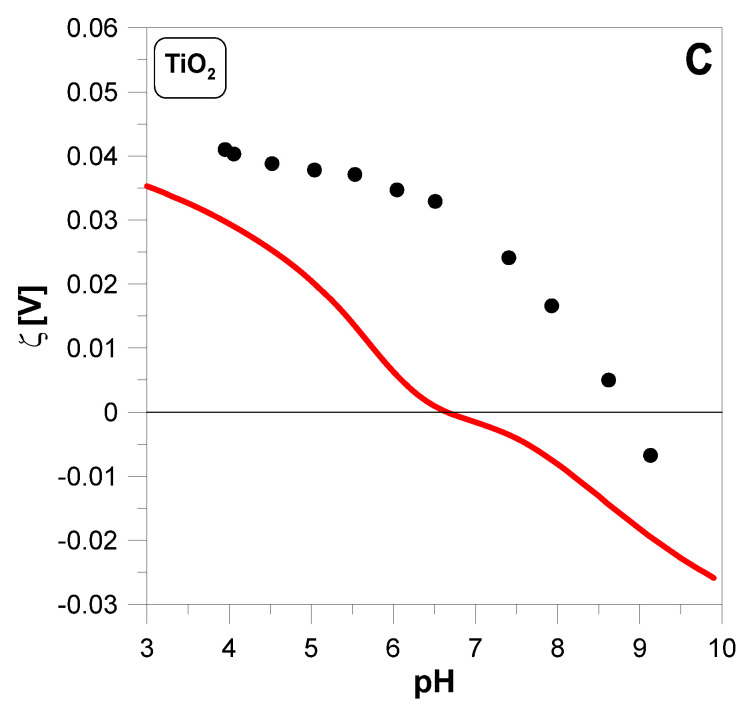
The adsorption edge (**A**) of $Cu^{2+}$ ions (initial concentration 1 mM) on $TiO_{2}$, the hydrogen surface charge density *σ_H_* (**B**), and the ζ-potential of $TiO_{2}$ particles (**C**) as a function of pH measured in 0.1 M $KNO_{3}$ solution containing 2 g·dm^−3^ oxide. Theoretical predictions (red lines) were based on surface reaction (1a–d) and (4a,b). The contributions from the surface species ${\left(SOH\right)}_{2}Cu{\left(OH\right)}_{2}$ (green line) and ${\left(SOH\right)}_{2}SOCu_{2}{\left(OH\right)}_{3}$ (blue line) are also shown. The values of the parameters used in the calculations are summarized in Table 1.

Figure 4 shows how the above complexes reproduce the data measured for $TiO_{2}$. The fit is not better than using the complexes described by Equation (3a,b). This is particularly visible for the electrokinetic potential. The measurement results clearly show that the ζ-potential is shifted towards positive values. This results from the adsorption of positively charged copper ions, which is considered by Equation (3a). On the other hand, Equation (4a,b) describe the $Cu^{2+}$ adsorption process so that the total surface charge does not change during the process, since the adsorption of copper ions is accompanied by the desorption of hydrogen ions with an equivalent charge.

This example shows that the electrokinetic potential measured during the adsorption of divalent ions can be a crucial factor in selecting the correct surface complex structure.

Above pH 6, copper ions can precipitate from the solution in the form of $Cu{\left(OH\right)}_{2}$ and accumulate on the oxide surface:

(5) $$Cu^{2+}+{2H}_{2}O\to Cu{\left(OH\right)}_{2}+2{\;H}^{+}$$

The equilibrium constant of this reaction is LogK=−8.67 [37]. To what extent the reaction contributes to the uptake of copper ions from the solution is difficult to determine based on the analyzed experimental data. This is because the stoichiometry of reaction (5) is the same as the reaction (3b)—here we have the same number of copper ions and protons involved. Additionally, in both cases, electrically neutral complexes are formed. To decide this, it would be necessary to examine metal oxides with adsorbed copper using EXAFS spectroscopy. Unfortunately, we do not have such instrumentation. However, such studies were performed by Peacock and Sherman [18], who studied the adsorption of $Cu^{2+}$ on iron oxides. They did not find the presence of $Cu{\left(OH\right)}_{2}$; therefore, we did not include reaction (5) in our modeling.

## 4. Materials and Methods

In our experiments, we used three commercial oxides provided by Evonik, Essen,

Germany. Titanium dioxide (Aeroxide P25) is hydrophilic fumed $TiO_{2}$ consisting of anatase and rutile with a weight ratio of 80:20. Its specific surface area is 50 ± 15 m^2^·g^−1^ (BET), and an average size of particles is about 21 nm. Aluminum oxide (Aeroxide Alu C) is $Al_{2}O_{3}$ consisting of γ and δ phases with a specific surface area of 100 ± 15 m^2^·g^−1^ and particle size 7–20 nm. Silica (Aerosil 200) is an amorphic $SiO_{2}$ with a specific surface area of 200 ± 25 m^2^·g^−1^ and the average size of particles is 14 nm. All oxides were used in adsorption experiments without further purification. Possible contamination of oxides with HCl coming from their synthesis can be eliminated during data processing as described in our previous paper [24].

The activity of ions in a solution depends on its ionic strength. Therefore, we must ensure that ionic strength is constant during ion adsorption measurements. The solution must contain a sufficiently high concentration of a neutral salt whose ions do not interact with the metal undergoing adsorption (e.g., forming stable complexes with it) or are not subject to specific adsorption on the oxide surface.

To maintain constant ionic strength during measurements we used an inert electrolyte solution $(KNO_{3}$) which was prepared from analytical grade salt. The solutions used in titration (0.1 M $HNO_{3}$ and 0.1 M KOH) were obtained from the analytical weights of the reagents. The stock solution of copper ions was prepared from $Cu{\left(NO_{3}\right)}_{2}\cdot 3H_{2}O$ analytical grade salt. To prepare these solutions we used Milli-Q quality water.

The Metrohm Titrando 907 device with two measuring interfaces and two 800 Dosino dosing units (with an acid and a base) was used for all potentiometric titrations. The Metrohm Tiamo 2.5 software was applied to control the entire titration process. The glass-jacketed stirring vessel was connected to the Julabo F12 thermostat to maintain the temperature of the solution at 25 °C. Pure argon was passed through the vessel to remove $CO_{2}$ traces. The combined glass electrode (Unitrode from Metrohm) was used to measure the pH. The fixed drift limit (0.5 mV·min^−1^) was set to save the final pH value in each titration step. The glass electrode was calibrated at pH 9, 7, and 4 using three buffer solutions.

The titration procedure was as follows. First, 100 cm^3^ of a solution containing 0.1 M $KNO_{3}$ and 1 mM $Cu^{2+}$ with 0.2 cm^3^ of 0.1 M $HNO_{3}$ was placed in the vessel; next, 0.2 g of oxide was added (to have the oxide concentration of 2 g·dm^−3^). Finally, we performed titration with 0.1 M KOH to a pH about 9. A more extensive presentation of all aspects of the titration of metal oxide suspensions can be found in the literature [38].

We calculated the hydrogen surface charge density σ_H_ from the below Equation (6) [27]:

(6) $$\sigma_{H}\left[\frac{C}{m^{2}}\right]=\frac{F}{A_{s}C_{s}}\left[\left(\frac{C_{A}{\cdot ∆V}_{A}}{V_{susp}}-\frac{1}{\gamma_{H^{+}}}10^{-pH}\right)-\left(\frac{C_{B}\cdot {∆V}_{B}}{V_{susp}}-\frac{1}{\gamma_{{OH}^{-}}}10^{pH-14}\right)\right]$$

where F—the Faraday constant [C·mol^−1^], $A_{s}$—the specific surface area [m^2^·g^−1^], $C_{s}$—the oxide concentration in the suspension [g·dm^−3^], $C_{A},C_{B}$—the concentration of $HNO_{3}$ and KOH solutions [M], ${∆V}_{A},{∆V}_{B}$—the added volume of $HNO_{3}$ and KOH [dm^3^], $\gamma_{H^{+}},\gamma_{{OH}^{-}}$—the activity coefficients of $H^{+}$ and $OH^{-}$ ions, $V_{susp}$—the oxide suspension volume [dm^3^].

The copper ion concentration was measured by direct potentiometry using a Cu-ISE with a crystal membrane (Metrohm No. 6.0502.140, Metrohm, Herisau, Switzerland) with a reference electrode (LL ISE Reference, Metrohm No. 6.0750.100). The measuring range of the Cu-ISE is from 1 × 10^−8^ M to 0.1 M, and the pH range from 2 to 12. Cu-ISE calibration was performed using three standards (2, 0.2, 0.02 mM $Cu^{2+}$) dissolved in 0.1 $KNO_{3}$ solution with 0.2 cm^3^ of 0.1 M $HNO_{3}$ (the volume of the standards was 100 cm^3^). The drift limit for the electrode was set at 0.5 mV·min^−1^.

The electrokinetic potential (ζ) of the oxide suspension as a function of pH was measured using a Malvern Zetasizer Nano ZS (Malvern Pananalytical, Malvern, UK). For this purpose, a small amount of suspension (approximately 0.3 cm^3^) was taken from the titration vessel after the pH and copper ion concentration readings had stabilized and then introduced into the high concentration zeta potential cell of the Zetasizer. After measuring the ζ-potential, the solution sample was returned to the titration vessel. The ζ-potential was calculated from electrophoretic mobility using the Smoluchowski equation [39].

## 5. Conclusions

The study of metal ion adsorption on oxides or other adsorbents should not be limited to measurements of the uptake of these ions from the solution but should also include a determination of other quantities that change with ion adsorption, such as solution pH and electric charge on adsorbent particles. However, the data obtained without their subsequent analysis using a model with solid thermodynamic foundations and considering the molecular mechanism of the ion adsorption process is a waste of the opportunity to gain insight into the detailed nature of this phenomenon. One model created to analyze ion adsorption on oxides is the 2-pK Triple Layer Model used in this work.

By using this model to analyze a set of data obtained for the adsorption of copper ions on three oxides, including the adsorption edge, the hydrogen surface charge, and the ζ-potential, we showed that the mechanism of $Cu^{2+}$ ion binding to the oxide surface can be determined in this way. The dominant complex is the surface species $SOCu\left(OH\right)$ with a small share of $SOCu^{+}$ (about 10%). The presence of the latter complex is particularly important for reproducing the ζ-potential.

The presented results do not definitively prove that copper ions are adsorbed on the surface of the tested oxides in the form of complexes described above. However, they confirm that even under conditions of high surface coverage, simple surface species can be found that allow for the correct reproduction of various experimental data.

## Figures and Tables

**Table 1:** Equilibrium constants of the surface reactions used in modeling experimental data.

	LogK		
Surface Reaction	$TiO_{2}$	$Al_{2}O_{3}$	$SiO_{2}$
$SOH+H^{+}\to SOH_{2}^{+}$	4.1	6.1	−0.1
$SOH\to SO^{-}+H^{+}$	−9.2	−11.5	−7.0
$SOH+K^{+}\to SO^{-}{\_K}^{+}+H^{+}$	−8.6	−10.7	−6.8
$SOH+H^{+}+NO_{3}^{-}\to SOH_{2}^{+}\_NO_{3}^{-}$	4.6	6.9	0.1
$SOH+Cu^{2+}\to SOCu^{+}+H^{+}$	−1.2	−1.8	−5.2
$SOH+Cu^{2+}+H_{2}O\to SOCu\left(OH\right)+{2H}^{+}$	−8.6	−9.8	−9.8
$2SOH+Cu^{2+}+2H_{2}O\to {\left(SOH\right)}_{2}Cu{\left(OH\right)}_{2}+2H^{+}$	−5.4		
$3SOH+2Cu^{2+}+3H_{2}O\to {\left(SOH\right)}_{2}SOCu_{2}{\left(OH\right)}_{3}+4H^{+}$	−11.8		

TiO_2_: N_s_ = 12.5 sites nm^−2^; c_1_ = 1.0 F·m^−2^, c_2_ = 1.0 F·m^−2^; Al_2_O_3_: N_s_ = 8.0 sites nm^−2^; c_1_ = 1.2 F·m^−2^, c_2_ = 1.2 F·m^−2;^ SiO_2_: N_s_ = 4.6 sites nm^−2^; c_1_ = 1.3 F·m^−2^, c_2_ = 0.1 F·m^−2^.

## Data Availability

Data are contained within the article.
